# Intention to Accept Pertussis Vaccination for Cocooning: A Qualitative Study of the Determinants

**DOI:** 10.1371/journal.pone.0155861

**Published:** 2016-06-02

**Authors:** Olga Visser, Jeannine L. A. Hautvast, Koos van der Velden, Marlies E. J. L. Hulscher

**Affiliations:** 1 Department of Primary and Community Care, Radboud University Medical Center, Nijmegen, the Netherlands; 2 Scientific Institute for Quality of Healthcare, Radboud University Medical Center, Nijmegen, the Netherlands; Universidad Nacional de la Plata, ARGENTINA

## Abstract

**Context:**

Several countries have reported a resurgence of pertussis in the last decades. This puts infants (especially <6 months) at risk of severe complications, because they are too young to be fully protected by vaccination. The global pertussis initiative has proposed pertussis vaccination of young infants’ close contacts, in order to reduce pertussis transmission and the burden of the disease on infants. Our aim is to explore the perceived determinants (barriers and facilitators) of intention to accept vaccination among the possible target groups of pertussis vaccination for cocooning. Consideration of these determinants is necessary to optimise the uptake of the vaccination.

**Methods:**

We conducted 13 focus groups and six individual semi-structured interviews with members of possible target groups for pertussis cocooning (i.e. parents, maternity assistants, midwives, and paediatric nurses) in the Netherlands. Here, both maternal pertussis vaccination as well as pertussis cocooning has not been implemented. The topic list was based on a literature review and a barrier framework. All interviews were transcribed verbatim and two researchers performed thematic content analysis.

**Findings:**

The participants’ risk perception, outcome expectations, general vaccination beliefs, moral norms, opinion of others, perceived autonomy, anticipated regret, decisional uncertainty, and perceived organisational barriers were all factors that influenced the intention to accept pertussis vaccination for cocooning.

**Discussion:**

This study has identified nine perceived determinants that influence the intention to accept pertussis cocooning vaccination. We add the following determinants to the literature: perceived cost-effectiveness (as a concept of outcome expectations), justice (as a concept of moral norms), anticipated regret, and decisional uncertainty. We recommend considering these determinants in vaccination programmes for pertussis cocooning vaccination. Experience, information and trust emerged as predominant themes within these determinants. These themes require particular attention in future research on vaccination acceptance, especially with regard to their role in use and implementation in policy and practice.

## Introduction

Pertussis is a dangerous disease for young infants. They suffer the greatest risk of severe complications and are too young to be fully protected by vaccination[1–3]. Despite longstanding vaccination programmes with high coverage, several countries have reported a resurgence of pertussis in the last decades[4–9]. This places infants at risk of pertussis infection[8,10].

To reduce the burden of pertussis for infants, some countries have introduced more targeted vaccination approaches to their childhood vaccination programmes. One of these approaches is cocooning[5,11–13]. In a pertussis cocooning strategy, a pertussis-containing vaccine, most often the combined tetanus toxoid, reduced diphtheria toxoid, and acellular pertussis (TDaP) is offered to those around a newborn. The aim is to prevent transmission to the baby. The target groups for cocooning vaccination include parents, close household contacts, and healthcare workers who take care of infants.

Given the international debate regarding the uptake of pertussis cocooning among intended recipients as well as on policy level, the expected acceptance of this strategy should be considered before implementation[14–17]. A well-planned implementation strategy is crucial to prevent an uptake problem and to ensure widespread acceptance. This strategy should be carefully linked to the relevant determinants of acceptance[18,19].

Multiple studies describe the determinants of accepting pertussis vaccination for cocooning, investigating both the intention to accept as actual acceptance in diverse target groups [20–41]. Wiley et al., for example, report that a healthcare provider’s recommendation, the belief that the vaccine is safe and effective, and the access to good information about pertussis correlate with pregnant women’s acceptance of postpartum pertussis vaccination in Australia[20]. Other studies involving paediatric healthcare professionals as well as parents also find that the perceived risk of pertussis, previous vaccination acceptance, and knowledge influence acceptance[21–41].

To identify possible determinants of acceptance, it is important to gain an in-depth understanding of the target groups’ values, opinions, behaviours, and social contexts regarding pertussis cocooning. The current evidence is mainly based on quantitative research. Therefore, this study qualitatively explores the perceived determinants (barriers as well as facilitators) of intention to accept pertussis cocooning vaccination in both parents and various groups of healthcare professionals in the Netherlands and it describes the differences between the groups that we interviewed.

## Methods

### Design

We conducted focus groups to explore all the relevant perceived determinants of intention to accept pertussis vaccination. A focus group encourages interaction between the participants, which facilitates a rich discussion[42]. If no focus group was possible due to organisational constraints, individual interviews were carried out.

### Study participants

We selected parents of newborn babies and three different subgroups of healthcare workers: maternity assistants, midwives, and paediatric nurses, as they reflect groups, which are in close and prolonged contact with newborns. The study was performed in the Netherlands, a country where both maternal pertussis vaccination as well as pertussis cocooning were not implemented during the study period. In the Netherlands governmentally advised vaccination programmes are normally paid for by the government, which was the assumption of the interviewees during the interviews.We organised homogeneous focus groups between May 2011 and June 2012.

#### Parents

We interviewed pregnant women as well as mothers and fathers of newborns to determine their opinions. We asked seven teachers of pregnancy exercises from three different institutions in three different geographical areas in the Netherlands to invite their current groups of antenatal and postnatal women to participate. Their partners were also invited for a separate focus group. The neighbourhoods of these pregnancy classes differed clearly with respect to socio-economic features. The teachers registered the names for participation and a convenient time and place for the focus groups was arranged.

#### Maternity assistants

One home care organisation invited as many maternity assistants as necessary from ten teams. These maternity assistants have completed intermediate vocational education and take care of the mother and child at their home in the first week after delivery. Focus groups were planned in the 1.5 hours before a planned team meeting. The home care organisation manager invited all their team members and registered the potential participants.

#### Midwives

One regional professional organisation of midwives invited all 35 of their midwives to participate. These community midwives work in private practices and take care of the uncomplicated births in the Netherlands, either at home or in a hospital. The midwives registered for participation by e-mailing the primary researcher, who arranged an appointment for the focus group.

#### Paediatric nurses

To obtain the opinions of nurses in regular close contact with infants, we chose to interview nurses working in the neonatal care unit of a Dutch university medical centre. The team managers sent invitations to 125 neonatal care nurses, who were asked to register by e-mailing the primary researcher. Since a focus group appeared impossible because there were too few reactions, we interviewed the individual participants.

### Data collection

The focus groups and interviews lasted around 1.5 hours, and a trained moderator (OV) and a research assistant (JW or LK) conducted them. We used a semi-structured interview guide, which was based on themes derived from the available literature and a barrier framework[18,24–26,43–46]. The same interview guide was used both for the focus groups as well as the interviews.

The interview topics included personal, external (social and societal), and organisational factors[18] We revised the interview guide after a pre-test. New themes that emerged during the focus groups and interviews were intuitively added to the interview guide by the researcher, to be verified in the next focus groups and interviews. New focus groups and interviews were planned for each group until no new concepts or themes emerged and theoretical saturation occurred.

Before enrolment, all participants had received a leaflet with information regarding the purpose of the focus group or interview, the voluntariness of participation, and a short introduction to the pertussis cocooning strategy. The focus groups and interviews started with an introduction to the study objectives and the role of the participants during the focus group, where the interactive character of the meeting was emphasised. The participants were assured that everything they said would be anonymous and confidential, and we asked their consent to record the focus group. Then, we introduced the key question for the focus group: ‘Why would you accept or refuse pertussis vaccination if it were offered to you in the context of a cocooning programme?’ We assured the participants that we were interested in arguments both for and against acceptance, and we asked them to write down these reasons for themselves first. An open group discussion followed, and the moderator asked more in-depth questions. The assistant checked whether all the topics taken from the interview guide had been covered and introduced a theme where relevant. In order to ascertain whether new arguments would arise for respondents, in case they would have access to more detailed information on pertussis and vaccination, we then handed out a sheet with factual information about pertussis and the proposed vaccination (comparable to information routinely provided in public health leaflets) to the participants. Then the group discussed this information.

### Analysis

The focus groups and interviews were recorded with a digital voice recorder and transcribed verbatim by an independent transcriber (RW). The moderator and a research assistant independently coded all the focus groups and interviews. They used the qualitative software programme Atlas.ti for this purpose. We performed a thematic content analysis after completion of all focus groups and interviews and extracted main themes by means of both an inductive approach and a deductive approach. The moderator and research assistant discussed the codes and themes. They discussed any disagreements further until they achieved consensus. This study adheres to the Coreq guidelines for reporting qualitative studies[47].

### Ethical review

The Medical Ethics Committee of the Arnhem—Nijmegen region assessed the study and concluded that it was exempt from their approval; reference number: 2010/475.

## Results

We conducted a total of 13 focus groups and six individual interviews. There were 21 new parents (five focus groups, 17 women and four men), 26 maternity assistants (five focus groups, all women), 12 midwives (three focus groups, all women), and six neonatal care nurses (individual interviews, all women). The focus groups consisted of a minimum of three participants and a maximum of seven participants.

The expected, or recently born, child of the interviewed parent was usually their first baby; only four of these parents already had children. The average work duration of the professionals was 10 years, ranging from less than a year (just started working) to 42 years.

**Fig 1:**
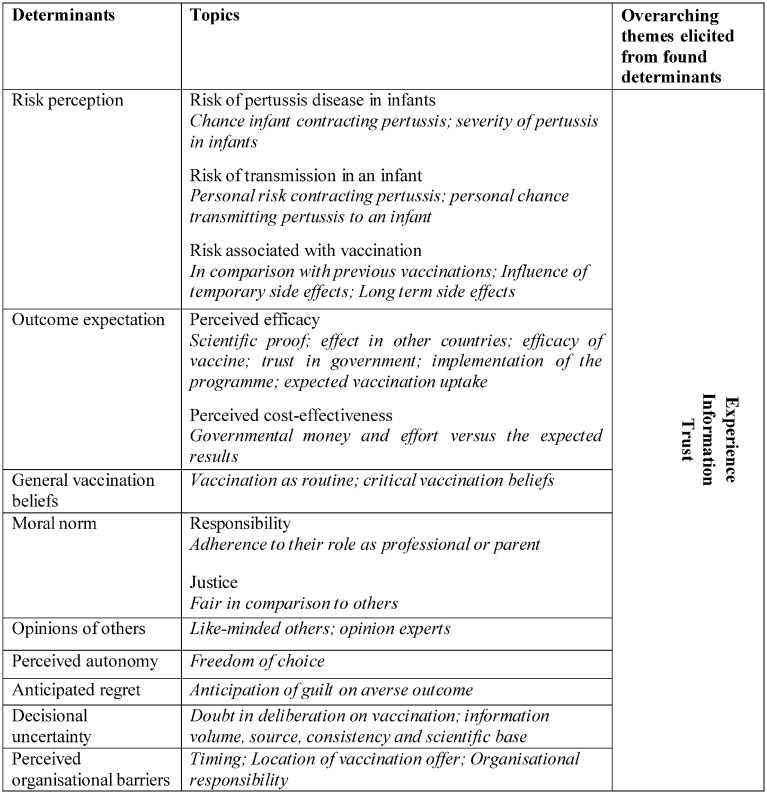
Positional map of determinants and overarching themes of parental intention to accept pertussis cocooning vaccination.

We found nine common factors all target groups considered in the decision on their own vaccination, that was intended to protect their baby or a patient. Some of the factors were more prominent in specific target groups. In the following these nine perceived determinants of intention to accept pertussis vaccination for cocooning will be described. In Fig 1 an overview of these determinants is presented, as well as the overarching themes which we elicited from these determinants. We elaborate on the overarching themes in the discussion section of this article.

### Risk perception

In deciding whether to accept pertussis vaccination, all groups evaluated their perception of three risks relative to the vaccination strategy: (1) the risk of pertussis disease for an infant, (2) the risk that they would transmit pertussis to an infant, and (3) their own risks due to vaccination. Within all participant groups, opinions differed on how to cope with risks, ranging from a risk-averse stance to the view that risks are an inevitable part of life.

#### Risk of pertussis disease for infants

In evaluating the risk of pertussis for infants, the participants considered both the chance that an infant would contract pertussis and the severity of the disease for infants. In all the groups, experience with infant pertussis in the direct environment seemed to influence the evaluations.

*Witnessing this child eventually only having some necrotized lung tissue left and actually dying*. *It made me realise that a simple disease like whooping cough can have major consequences for these little ones*.

–Neonatal care nurse

Though participants divided over all groups, believed the risk of pertussis to vary for infants, we observed that this belief was most expressed by the professionals. This seemed to mitigate the sense of urgency they felt in relation to their own need of vaccination. For example, they asked if severe pertussis occurs more often in specific groups, such as non-breast-fed children. For them, attributing the risk of pertussis to a specific group with a specific behaviour seemed to minimise the risk for the rest of the children, which then made vaccination less important. Furthermore, several participants from all target groups placed the risk of infant pertussis in the context of the total burden of all disease on infants and questioned the priority of pertussis: is it currently the most important disease for a prevention programme?

#### Risk of transmission to an infant

In assessing whether they might transmit pertussis to an infant, the participants reported two important factors. First, they appraised the risk of contracting pertussis themselves. Experience with adult pertussis in their environment and the view of their own health and immunity status seemed to influence their perception of this risk.

*I think my chances of contracting whooping cough are not very high*. *I figure my body can handle it*. *I'm healthy enough*.

–Maternity assistant

Second, participants assessed the chance of transmitting pertussis to an infant once they had contracted the disease themselves. This came forward most clearly in the interviews with the healthcare professionals. They looked for quantification of this risk and asked what evidence there was that they, as a profession, contributed to transmitting pertussis to infants. A few midwives felt that the nature of their work requires little contact with infants, and they therefore perceived the chance of their transmitting pertussis to an infant as small. Other professionals believed that other means of prevention—such as quick diagnostics or hygiene measures—would be enough to prevent transmission, i.e. a low risk. For parents, the chance of transmission was a less important issue in their considerations on pertussis vaccination acceptance, as compared to healthcare workers.

*On the other hand*, *sanitary regulations are so strict that we are constantly washing our hands*. *I wonder what the chances of infection are when you adhere to the hygienic regulations*.

–Maternity assistant

#### Risk associated with vaccination

The participating groups assessed the personal risks due to their own pertussis vaccination. From all target groups there were some participants, who compared the possible side effects to the side effects of previous vaccinations, and most assumed that this risk was low.

*I don't really see the problem*. *I'm not fond of injections*, *but come on*, *get it over with*, *have it*, *and you're done*. *You might have a bit of fever and a sore spot for a little while*, *but that's it*.

–Neonatal care nurse

Both parents as well as health care workers needed more information about the consequences of the temporary side effects. However they did differ in considering the context of these consequences: for instance, parents asked specific questions about the effects in the postpartum period, such as the effect on breastfeeding or the risk of contracting pertussis from the vaccination and transmitting it to their newborn. Professionals asked whether they could continue working after pertussis vaccination. Some participants of all target groups also focused on the long-term side effects. They felt they could not assess the risk of long-term side effects because the vaccines had not yet existed long enough. They compared this to the diethylstilbestrol (DES) treatment in the mid-20th century, which turned out to have long-term side effects nobody expected. Furthermore, some neonatal care nurses worried about antimicrobial resistance to vaccines and what that would mean for the future.

*I mean*, *the DES hormones situation way back when*, *we didn't know anything about that either and we used it anyway*. *Nobody knew it would get so badly out of hand*.

- Maternity assistant

### Outcome expectations

The expectations participants had about the outcomes of the cocooning strategy were important in forming opinions. This was reflected in how they perceived the efficacy of the programme, as well as the expected benefits versus the expected costs.

#### Perceived efficacy

To assess the efficacy, the participants asked themselves: how effective will cocooning be in preventing pertussis in infants? In considering their answers, the participants repeatedly said that the state of the scientific work proving the intervention would be effective was crucial for forming an opinion. This stood out especially in the opinions of the midwives, although all the participating groups said that the evidence base for the strategy was important.

How the participants perceived the efficacy of cocooning seemed to be shaped by (1) the effect of cocooning in other countries, (2) the perceived efficacy of the vaccine itself, (3) the participants’ trust in the government, (4) their opinions of the extent of the cocooning programme (i.e. which target groups would be included), and (5) future vaccination uptake. For example, some participants divided among all target groups argued that vaccination in general no longer seems to be a matter of course. Now it is difficult to close a circle of protection around an infant, which diminishes the potential effect of cocooning. In all different groups, others stated that the government does not take their responsibility for vaccination advice lightly. Therefore, if the government advised pertussis vaccination, they would trust it as an effective strategy.

*And the usefulness*. *I would like the results to be examined 10 years later to see whether it was any use vaccinating everybody and whether these numbers will have indeed decreased*.

–Midwife

#### Perceived cost-effectiveness

To evaluate the cost-effectiveness, the participants questioned whether the benefits would outweigh the money and effort the government had to invest. This question came forward in all the groups, but the midwives stated it most specifically. Others (from all groups) said that the efficacy should be optimal to achieve a balance, and they believed the whole population should be re-vaccinated.

*It may sound harsh*, *but all this to save one baby*. *Consider the costs and the system that has to be set up*. *Macro-economically*, *this makes you wonder whether it is really worthwhile*.

–Parent

### General vaccination beliefs

The opinions of vaccination in general are reflected in the evaluation of pertussis vaccination in the context of cocooning. Some participants stated that vaccination in general is routine; for them, it is just the normal thing to do. They trusted the government or science to prescribe a vaccination only if it was unquestionably ‘a good idea’. Not all people for whom the National Immunisation Programme (NIP) was routine automatically thought pertussis cocooning was routine as well. Mainly parents expressed this view.

*If people (i*.*e*. *government employees*, *OV) have come up with this solution*, *I guess it will be all right*.

–Parent

Further, other participants had rather critical beliefs about vaccination in general. Their arguments belonged to three groups. First, they criticised the necessity of new vaccines. They asked themselves: ‘where will it all end?’ They wondered if cocooning just reflected a lobby of the pharmaceutical industry promoting the use of a new vaccine. Second, the participants were suspicious of vaccination in the sense that it was ‘not natural’. Some perceived a vaccination as ‘rubbish that is injected into your body’. Third, some participants claimed that naturally overcoming an infection would improve their health. Notably, most respondents with this critical mindset were professionals.

*Isn't it supported by an enormous politically motivated group*, *or a pharmacological group that has earned lots of money with it*?

–Neonatal care nurse

### Moral norm

While evaluating their intention to accept pertussis vaccination for cocooning if it were offered to them, the participants’ moral norms seemed to influence them. They gave arguments about their feelings of responsibility and the justification of the programme.

#### Responsibility

Both professionals and parents said they felt a responsibility towards the infant at risk, and they took this into account when deciding about vaccination. Some professionals related this responsibility to their roles as professionals and said they would accept vaccination as a part of their profession. In contrast, others said that vaccination belonged to the personal domain, and they refused to accept it as a part of their profession. Some parents also felt obliged to set an example by accepting vaccination. They asked themselves: ‘If we don’t accept vaccination, why would others?’

*And for the family too*. *I don't think it looks very professional if you are the one who infects the child*…. *It is our priority to protect the child*… *Being there to take care of the child and at the same time infecting it would be rather contradictory*.

–Maternity assistant

#### Justice

In considering whether to accept a possible future pertussis vaccination, the respondents (mainly the professionals) said that they needed to feel that being asked to accept vaccination was fair. Some asked themselves if they thought it fair that they had to take responsibility for the health of a baby by getting vaccinated if parents, in their eyes, did not act responsibly (if they refused vaccination themselves, smoked, or bottle-fed the newborn). Other respondents said it was unfair that healthcare professionals were ‘always’ the ones who had to be vaccinated.

*To what extent*? *Should I have the vaccination when the mother chooses to smoke and to bottle-feed the child*? *That is my argument*. *I have to work hard (to keep the baby safe) and she doesn't care at all*.

–Midwife

### Opinions of others

While some participants said they would decide about vaccination by themselves, others clearly stated that they would value discussion with like-minded people before deciding. Yet all the participants were conscious of other people’s opinions of vaccination. Professionals and parents alike tended to value the opinions of people with the relevant medical expertise. The opinions of family and friends were also important to parents. The professionals also said their colleagues influenced their opinions. However, maternity assistants were an exception: they said their profession was so solitary that they hardly had a chance to discuss such topics with their colleagues.

*Among colleagues we sometimes talk about things during a break*. *One knows more about this and another about that*, *so eventually your decisions are better reasoned*. *At least you know for a fact that you have really given it some thought*.

–Neonatal care nurse

### Perceived autonomy

In the interviews, all the participants discussed the need to critically appraise their opinions of pertussis vaccination themselves. Some stated that the right to choose freely should not always prevail because the negative consequences of some choices could harm others. Nonetheless, such a restriction on their own choices led most participants to reject the offer of vaccination. This was most explicit among the maternity assistants when they recounted their experience with the Pandemic 2009H1N1 influenza. The pressure from their employers to accept vaccination negatively influenced their decision then and would do so again in the future.

*Yes*, *mainly because it is very important to decide about your own body*… *after all*, *despite the best intentions*, *if people cannot choose for themselves*, *they'll object by saying*: *“Hey it is my body you are injecting*.

–Neonatal care nurse

### Anticipated regret

Another influential factor in considering pertussis vaccination is the possibility of a future consequence of their decision that the participants would regret. They mainly referred to the possible consequences of not being vaccinated. If a child in their environment then contracted pertussis, they would seriously regret it and feel guilty about not accepting pertussis vaccination. This argument emerged in all the groups. The midwives added an extra dimension to this argument: they would also regret losing income from their private practices if they became ill themselves. Some participants would also regret accepting the vaccination if they suffered physical problems at some point in the future that they thought the vaccination caused.

*Of course it’s terrible when a child falls ill*, *especially a very small child*, *but if you knew you could have prevented it*, *you'd never forgive yourself*.

–Parent

### Decisional uncertainty

The amount of doubt participants showed in their deliberation about accepting the vaccination seemed to influence the decision. Some participants had confidence in their own opinion because of their education or their trust in the governmental organisations that provided the guidelines. Others believed they should be able to make an educated decision, but felt inadequately equipped to do so. When the participants tried to explain their uncertainty, they said it seemed to have become more difficult to handle the information for their decision because more and more information—often with contradictory messages—had become available. They then tried to verify the information and take into account the objectivity and reliability of the source and the evidence base, as well as the consistency of the message coming from different sources. Referring to the Pandemic 2009H1N1 influenzain 2009, the participants said that the more unrest they perceived in society and the more often messages appeared in the media, the less trust they had and the more difficult their decision would be. Both parents and healthcare workers recognised their decisional uncertainty.

*What bothers me most was that both websites boast scientific underpinning*. *What I would like is one objective website because now one of them is for and the other is against*, *and how are you supposed to compare the two*? *Flip a coin*?

–Parent

### Perceived organisational barriers

Although the location, time, and vaccine provider of the future pertussis vaccination programme seemed to influence all the groups, they thought other issues were the most important ones.

Some parents believed it would be best if mothers were re-vaccinated before pregnancy, but at the same time they questioned the practical implications of this advice. The concept of vaccinating the mother right after birth caused some specific concerns about the vaccination location. They said that having to go to a specific health clinic for vaccination would reduce their willingness to accept. For them, the vaccine provider was a lesser issue; combining the vaccination with an already standard appointment seemed more important.

For neonatal care nurses and maternity assistants, the vaccine would be most logically provided via the occupational health service. However, community midwives who work in their own private practices with busy shift schedules often have no arrangements with an occupational health service. If the organisation around administering a vaccination were their own responsibility, that would hinder acceptance. All the professionals stated that an easily accessible location for vaccination with flexible timing options would facilitate their acceptance.

*Logistically*, *things have not been arranged*, *things like where to get it since we're not a group*. *In an institution or an organisation you can apply to your manager or your work regulations*. *But we are self-employed*, *so where do we go*? *We can go see our general practitioner and pay*.

–Midwife

## Discussion

### Determinants of pertussis cocooning acceptance

In this study, we have identified nine perceived determinants of intention to accept pertussis cocooning among different target groups in the Netherlands. Five of these determinants (i.e. risk perception, general vaccination beliefs, the opinions of others, perceived autonomy and organisational issues) have been previously identified in studies of pertussis cocooning acceptance [20–41], albeit in a slightly different form. We have added perceived cost-effectiveness (as a concept of outcome expectations), justice (as a concept of moral norms), anticipated regret, and decisional uncertainty as perceived determinants of the intention to accept pertussis cocooning.

#### Relation to known determinants of pertussis cocooning acceptance

All studies of the determinants of pertussis cocooning acceptance, both on the intention to accept as the actual acceptance, name the influence of the risk perception of pertussis[20–41], which is in line with our findings. It is also consistent with Brewer’s meta-analysis[48], which confirms the role of the perceived risk of vaccine-preventable diseases in relation to vaccination behaviour in general. The studies of pertussis cocooning acceptance also mention some form of the perception of the risk of transmission to an infant and the risk associated with vaccination[20–26,29,30,36–39]. However, most of these studies hardly distinguish them as different risks that are weighed in decision-making. They appear mainly in overall categories such as ‘fear of vaccination’. A study ofHayles et al also suggests that the perceived risk of transmission to an infant is an important factor in postpartum pertussis vaccine acceptance among mothers and demonstrates that concerns on vaccine safety and efficacy relate to non-acceptance [37].

The general vaccination beliefs comprise of separately described arguments that now appear increasingly more often in the vaccination acceptance literature. These arguments include consideration on vaccination and naturalistic beliefs and seem to be formed by trust in government, science and industry[21,22,24,49–51]. However, our data supports the idea that these beliefs are part of the same context: the general opinion on vaccination.

Most participants valued the opinions of medical experts while deciding for or against vaccination. This is consistent with other studies of the acceptance of pertussis cocooning vaccination, which report the importance of healthcare providers’ recommendations as an influential factor[20,22,26,29,31,33,36–38,40,41].

Furthermore, some studies have reported the influence of perceived autonomy in the healthcare workers’ decisions about accepting vaccination for pertussis and for influenza[21,29,52–54]. Parents’ ‘right to choose yourself’ has previously been described in studies of the acceptance of measles, mumps, and rubella (MMR) vaccine[55].

Finally, our study underlines the importance of organisational factors in the implementation of a vaccination programme[18,29,31,36,38,39,43,45]. Importantly, different target groups have different preferences. The absence of available infrastructure for administering vaccination is seen as difficult, especially for the target groups of new parents and midwives. Healy et al. (2011) argue that this is also an important barrier to accepting pertussis cocooning vaccination in the USA[56]. Although this makes the organisation of a vaccination programme for pertussis cocooning challenging, our data provide suggestions regarding vaccine delivery that may help mitigate barriers to vaccine completion by target groups, which would help those aiming to minimise the negative influence that some organisational factors have on acceptance.

#### What this study adds

Although other studies describe outcome expectations as an influential factor[20–26,30,34,36–38] our participants, interestingly, also said that their perceived cost-benefit ratio (of a government vaccine strategy) influenced their decision. This suggests that people may weigh societal benefits and drawbacks for their personal decisions. To our knowledge, this has not previously been described in the vaccination acceptance literature.

In the literature about pertussis cocooning vaccination acceptance and vaccination acceptance in general, the concept of moral norm has been emphasised with regard to responsibility. Sometimes explicitly[36,57–60], sometimes implicitly; ‘to protect others’ and ‘to protect my patient’ are the phrases used as important reasons for accepting a vaccine[21,23,25,26,37,43,49]. This partially concurs with our findings.Our data suggest the possible addition of justice to the concept of the moral norm. Since ‘justice’ was more frequently a topic for professionals, it may function differently in different vaccination settings.

Anticipated regret has not yet appeared in other research about accepting vaccination for pertussis cocooning. However, recent research in other settings seems to show that anticipated regret does indeed influence vaccination decisions [61–64].

Miller’s study states that ‘a lack of information to base a decision on’ was a reason for people to refuse pertussis vaccination in the USA[22]. This may be similar to the decisional uncertainty found in our data. However, some participants declared that this decisional uncertainty originated in the large amount of available information about vaccination that propagates strong opinions either for or against. This resembles Poltorak’s description of parents’ uncertainty in deciding whether to accept an MMR vaccination for their child[55].

### Experience, information, and trust

In reflecting on our data, we noticed three predominant themes rooted in the different determinants of intention to accept pertussis vaccination: experience, information, and trust. These themes might be important in understanding the decision-making for pertussis cocooning vaccination, and they might provide entry points for the design of an effective vaccination programme.

Participants’ experiences are important in their assessment of the various risks for their decision–making, and these experiences are also important in formulating the roles of autonomy and decisional uncertainty. In previous studies associations have been described between earlier vaccine acceptance (for instance for influenza) and present acceptance of a pertussis cocooning vaccination [23,27,28,32]. Although a person’s experiences are, of course, inalterable, their importance in decision-making might justify further study of how experience can help in interventions for encouraging acceptance of vaccination.

Studies for improving the acceptance of vaccination often recommend attuning information to the needs of target groups[43,45,65]. In line with these findings, most study participants indeed needed information; they asked many questions before they formed an opinion about vaccination for pertussis cocooning. In search for answers, they reported to value information based on the source, volume, consistency and scientific base. Knowing what information to trust seemed to be a difficult task, as some participants stated that their search for answers resulted in decisional uncertainty. Notably, part of our participants also stated they perceived public health information biased in favour of vaccination promotion, which added to their uncertainty. This could be understood to be a result of their awareness of the double role a government has in voluntary vaccination programs: to stimulate vaccination acceptance, while respecting and fostering the public’s autonomous choice on vaccination [66]. It also fits with the societal change in trust described in the next paragraph. Moreover, it suggests that the power of public health authorities to improve vaccination acceptance through information provision might be restricted.

Other studies in which uncertainty appeared report that providing extra, attuned information had limited effects on vaccination acceptance[67,68]. Some authors have even questioned whether information that provides arguments for choosing leads to a decision at all[69]. Thus, the role of information in decision-making in vaccination acceptance is controversial. Therefore, attention should be given to alternatives for information provision in vaccination programmes aimed at optimising acceptance.

Trust is currently a more often debated issue in the literature about vaccination acceptance[49,70,71]. In our findings, the importance of trust is reflected in its influence on the acceptance of pertussis vaccination via the general vaccination beliefs, perceived efficacy, and decisional uncertainty. Trust in government, science, and industry positively influenced acceptance because some participants believed that a vaccination would only be advised if positive outcomes were expected. In contrast, some participants formulated what we called ‘critical vaccination beliefs’ in which distrust was very important. This corresponds with the changes in society toward a risk culture in which manufactured risks seem exceedingly important and lead to the distrust of government, industry, and science[72,73] Such a culture has been described in the sociological literature[49,74–76]. An answer to this trend is not easily found, but we need to give it specific attention in future research about vaccination acceptance.

### Differences between target groups

Notably, most determinants were brought forward by parents as well as professionals and were generally comparable in the different groups. Nonetheless, specific groups emphasised certain determinants.

The most remarkable differences were noted between the parents and the healthcare professionals, especially for moral norms, both responsibility and justiceMost parents said they could see vaccination as a part of their responsibility for their child’s health. Professionals, however, saw vaccination having an impact on their personal lives; therefore, they refused to regard it as part of their professional responsibility. This is compatible with Baron-Epel’s qualitative study of pertussis cocooning vaccination[21]

Furthermore, the healthcare professionals most clearly expressed critical vaccination beliefs, doubts about the risks for the vulnerable infants for whom the programme is designed, and doubts about the expected efficacy of the programme. It is remarkable that the healthcare professionals, who have more knowledge about medicine and science, held more beliefs that seemed to contrast with this background. However, this is in line with studies that identify higher educated parents and healthcare professionals as risk groups for vaccine refusal[77–79] Pereti-Watel[74] offers an explanation for this phenomenon: the educated middle class is more hesitant about accepting vaccination on the basis of distrust. They seem to know enough to recognise ‘manufactured risks’, but too little to discard them as illegitimate science.

### Strengths and limitations

This study has some strengths and limitations that should be mentioned. First, it provides insight into a broad range of influencing factors that affect the intention to accept vaccination for pertussis cocooning in the Netherlands. We interviewed parents and healthcare professionals in four differing but relevant target groups, who have close and prolonged contact with newborns. Therefore, our data reflect a comprehensive range of determinants. This is one of the few studies to report a perspective of healthcare professionals as recipients of pertussis cocooning vaccination (rather than providers) Regrettably, we were able to conduct only individual interviews with the neonatal care nurses. However, the influence this had on the results appears limited, since no new themes emerged in these interviews. There could have been some selection bias: people with strong opinions about the subject might have been more inclined to enter the study. However, we met both advocates and opponents of vaccination in the focus groups and interviews. As we sampled parents from different socio-economic settings, we trust that most opinions possibly related to socio-economic background were covered. For this reason, we are confident that we have covered the broad array of the target groups’ arguments, both for and against vaccination. As organisation of health care varies between countries, our findings on organisational barriers may be not generalizable for other countries.

Second, the qualitative research method has the advantage of obtaining in-depth understanding of the factors in question. The inclusion of new participants until data saturation was achieved and the number of individual interviews and focus groups ensures good validity of the data.

## Conclusion

This study provides an indication of which influencing factors should be optimised to effectively implement pertussis cocooning in different target groups, or adapt a pertussis cocooning programme where it already exists. It provides perceived determinants of the intention to accept a pertussis cocooning vaccination for parents as well as for paediatric nurses, maternity assistants and midwives and is of relevance especially in countries in which pertussis vaccination is not mandated for these groups. These results need quantification for target groups and settings which have not yet been studied. The heterogeneity of our study’s determinants demonstrates the complexity of people’s decision-making about vaccination for pertussis cocooning. Experience, information, and trust are the predominant themes that emerged from the described determinants. These themes require particular attention in future research on vaccination acceptance, especially with regard to their role in use and implementation in policy and practice.

## References

[1] Greenberg DP, von Konig CH, Heininger U (2005) Health burden of pertussis in infants and children. Pediatr Infect Dis J 24: S39–43. 15876922 10.1097/01.inf.0000160911.65632.e1

[2] Winter K, Harriman K, Zipprich J, Schechter R, Talarico J, et al (2012) California pertussis epidemic, 2010. J Pediatr 161: 1091–1096. 10.1016/j.jpeds.2012.05.041 22819634

[3] Heininger U, Weibel D, Richard JL (2014) Prospective nationwide surveillance of hospitalizations due to pertussis in children, 2006–2010. Pediatr Infect Dis J 33: 147–151. 10.1097/01.inf.0000435503.44620.74 24413406

[4] Miller E (2014) WHO SAGE pertussis working group Background paper SAGE April 2014.

[5] Burns DL, Meade BD, Messionnier NE (2014) Pertussis resurgence: perspectives from the Working Group Meeting on pertussis on the causes, possible paths forward, and gaps in our knowledge. J Infect Dis 209 Suppl 1: S32–35. 10.1093/infdis/jit491 24626870

[6] Amirthalingam G (2013) Strategies to control pertussis in infants. Arch Dis Child 98: 552–555. 10.1136/archdischild-2012-302968 23698594

[7] de Greeff SC, de Melker HE, van Gageldonk PG, Schellekens JF, van der Klis FR, et al (2010) Seroprevalence of pertussis in The Netherlands: evidence for increased circulation of Bordetella pertussis. PLoS One 5: e14183 10.1371/journal.pone.0014183 21152071 PMC2995730

[8] McIntyre P, Wood N (2009) Pertussis in early infancy: disease burden and preventive strategies. Curr Opin Infect Dis 22: 215–223. 10.1097/QCO.0b013e32832b3540 19395958

[9] Celentano LP, Massari M, Paramatti D, Salmaso S, Tozzi AE, et al (2005) Resurgence of pertussis in Europe. Pediatr Infect Dis J 24: 761–765. 16148840 10.1097/01.inf.0000177282.53500.77

[10] van der Maas NA, Mooi FR, de Greeff SC, Berbers GA, Spaendonck MA, et al (2013) Pertussis in the Netherlands, is the current vaccination strategy sufficient to reduce disease burden in young infants? Vaccine 31: 4541–4547. 10.1016/j.vaccine.2013.07.060 23933365

[11] Van der Maas N (2014) Kinkhoestsurveillance in 2013 en 2014. Netherlands: Rijksinstituut voor Volksgezondheid en Milieu (RIVM).

[12] Chiappini E, Stival A, Galli L, de Martino M (2013) Pertussis re-emergence in the post-vaccination era. BMC Infect Dis 13: 151 10.1186/1471-2334-13-151 23530907 PMC3623740

[13] de Greeff SC, Mooi FR, Westerhof A, Verbakel JM, Peeters MF, et al (2010) Pertussis disease burden in the household: how to protect young infants. Clin Infect Dis 50: 1339–1345. 10.1086/652281 20370464

[14] Williams WW (2014) Noninfluenza Vaccination Coverage Among Adults—United States, 2012. 95–102 p. PMC4584647 24500288

[15] Urwyler P, Heininger U (2014) Protecting newborns from pertussis—the challenge of complete cocooning. BMC Infect Dis 14: 397 10.1186/1471-2334-14-397 25037057 PMC4223593

[16] Leboucher B, Sentilhes L, Abbou F, Henry E, Grimprel E, et al (2012) Impact of postpartum information about pertussis booster to parents in a university maternity hospital. Vaccine 30: 5472–5481. 10.1016/j.vaccine.2012.06.071 22771510

[17] Forsyth KD, Wirsing von Konig CH, Tan T, Caro J, Plotkin S (2007) Prevention of pertussis: recommendations derived from the second Global Pertussis Initiative roundtable meeting. Vaccine 25: 2634–2642. 17280745 10.1016/j.vaccine.2006.12.017

[18] Grol R (2013) Improving patient care: the implementation of change in health care. Chichester, England: Wiley-Blackwell 1 online resource (xvii, 374 s.) p.

[19] Grimshaw JM, Thomas RE, MacLennan G, Fraser C, Ramsay CR, et al (2004) Effectiveness and efficiency of guideline dissemination and implementation strategies. Health Technol Assess 8: iii–iv, 1–72. 10.3310/hta8060 14960256

[20] Wiley KE, Massey PD, Cooper SC, Wood N, Quinn HE, et al (2013) Pregnant women's intention to take up a post-partum pertussis vaccine, and their willingness to take up the vaccine while pregnant: a cross sectional survey. Vaccine 31: 3972–3978. 10.1016/j.vaccine.2013.06.015 23777954

[21] Baron-Epel O, Bord S, Madjar B, Habib S, Rishpon S (2012) What lies behind the low rates of vaccinations among nurses who treat infants? Vaccine 30: 3151–3154. 10.1016/j.vaccine.2012.02.074 22446637

[22] Miller BL, Kretsinger K, Euler GL, Lu PJ, Ahmed F (2011) Barriers to early uptake of tetanus, diphtheria and acellular pertussis vaccine (Tdap) among adults-United States, 2005–2007. Vaccine 29: 3850–3856. 10.1016/j.vaccine.2011.03.058 21459173

[23] Top KA, Halperin BA, Baxendale D, MacKinnon-Cameron D, Halperin SA (2010) Pertussis immunization in paediatric healthcare workers: knowledge, attitudes, beliefs, and behaviour. Vaccine 28: 2169–2173. 10.1016/j.vaccine.2009.12.060 20056190

[24] Cheng PJ, Huang SY, Shaw SW, Kao CC, Chueh HY, et al (2010) Factors influencing women's decisions regarding pertussis vaccine: A decision-making study in the Postpartum Pertussis Immunization Program of a teaching hospital in Taiwan. Vaccine 28: 5641–5647. 10.1016/j.vaccine.2010.05.078 20600516

[25] Wicker S, Zielen S, Rose MA (2008) Attitudes of healthcare workers toward pertussis vaccination. Expert Rev Vaccines 7: 1325–1328. 10.1586/14760584.7.9.1325 18980536

[26] Goins WP, Schaffner W, Edwards KM, Talbot TR (2007) Healthcare workers' knowledge and attitudes about pertussis and pertussis vaccination. Infect Control Hosp Epidemiol 28: 1284–1289. 17926280 10.1086/521654

[27] Tuckerman JL, Collins JE, Marshall HS (2015) Factors affecting uptake of recommended immunizations among health care workers in South Australia. Hum Vaccin Immunother 11: 704–712. 10.1080/21645515.2015.1008886 25715003 PMC4514246

[28] Lu PJ, Graitcer SB, O'Halloran A, Liang JL (2014) Tetanus, diphtheria and acellular pertussis (Tdap) vaccination among healthcare personnel-United States, 2011. Vaccine 32: 572–578. 10.1016/j.vaccine.2013.11.077 24308960 PMC5822443

[29] MacDougall DM, Halperin BA, MacKinnon-Cameron D, Li L, McNeil SA, et al (2015) The challenge of vaccinating adults: attitudes and beliefs of the Canadian public and healthcare providers. BMJ Open 5: e009062 10.1136/bmjopen-2015-009062 26419683 PMC4593142

[30] Vasilevska M, Ku J, Fisman DN (2014) Factors associated with healthcare worker acceptance of vaccination: a systematic review and meta-analysis. Infect Control Hosp Epidemiol 35: 699–708. 10.1086/676427 24799647

[31] Beel ER, Rench MA, Montesinos DP, Mayes B, Healy CM (2013) Knowledge and attitudes of postpartum women toward immunization during pregnancy and the peripartum period. Hum Vaccin Immunother 9: 1926–1931. 10.4161/hv.25096 23782490 PMC3906358

[32] Bodeker B, Walter D, Reiter S, Wichmann O (2014) Cross-sectional study on factors associated with influenza vaccine uptake and pertussis vaccination status among pregnant women in Germany. Vaccine 32: 4131–4139. 10.1016/j.vaccine.2014.06.007 24928791

[33] Clarke M, Thomas N, Giles L, Marshall H (2015) Community awareness and predictors of uptake of pertussis booster vaccine in South Australian adults. Vaccine 33: 7337–7343. 10.1016/j.vaccine.2015.10.068 26514422

[34] Dempsey AF, Brewer SE, Sevick C, Pyrzanowski J, Mazzoni S, et al (2015) Tdap vaccine attitudes and utilization among pregnant women from a high-risk population. Hum Vaccin Immunother: 1–7. 26430729 10.1080/21645515.2015.1094594 PMC4962928

[35] Donnan EJ, Fielding JE, Rowe SL, Franklin LJ, Vally H (2013) A cross sectional survey of attitudes, awareness and uptake of the parental pertussis booster vaccine as part of a cocooning strategy, Victoria, Australia. BMC Public Health 13: 676 10.1186/1471-2458-13-676 23875762 PMC3726505

[36] Hayles EH, Cooper SC, Sinn J, Wood N, Leask J, et al (2016) Pertussis vaccination coverage among Australian women prior to childbirth in the cocooning era: a two-hospital, cross-sectional survey, 2010 to 2013. Aust N Z J Obstet Gynaecol. 10.1111/ajo.12429 26751804

[37] Hayles EH, Cooper SC, Wood N, Sinn J, Skinner SR (2015) What predicts postpartum pertussis booster vaccination? A controlled intervention trial. Vaccine 33: 228–236. 10.1016/j.vaccine.2014.10.074 25444794

[38] O'Leary ST, Pyrzanowski J, Brewer SE, Barnard J, Beaty B, et al (2015) Influenza and Pertussis Vaccination Among Pregnant Women and Their Infants' Close Contacts: Reported Practices and Attitudes. Pediatr Infect Dis J 34: 1244–1249. 10.1097/INF.0000000000000873 26322660

[39] Rossmann Beel E, Rench MA, Montesinos DP, Healy CM (2014) Acceptability of immunization in adult contacts of infants: possibility of expanding platforms to increase adult vaccine uptake. Vaccine 32: 2540–2545. 10.1016/j.vaccine.2014.03.056 24681227

[40] Suryadevara M, Bonville CA, Cibula DA, Valente M, Handel A, et al (2014) Pertussis vaccine for adults: Knowledge, attitudes, and vaccine receipt among adults with children in the household. Vaccine 32: 7000–7004. 10.1016/j.vaccine.2014.10.018 25454869

[41] Wong CY, Thomas NJ, Clarke M, Boros C, Tuckerman J, et al (2015) Maternal uptake of pertussis cocooning strategy and other pregnancy related recommended immunizations. Hum Vaccin Immunother 11: 1165–1172. 10.1080/21645515.2015.1019188 25874807 PMC4514360

[42] Kitzinger J (1995) Qualitative research. Introducing focus groups. BMJ 311: 299–302. 7633241 10.1136/bmj.311.7000.299 PMC2550365

[43] Hollmeyer HG, Hayden F, Poland G, Buchholz U (2009) Influenza vaccination of health care workers in hospitals—a review of studies on attitudes and predictors. Vaccine 27: 3935–3944. 10.1016/j.vaccine.2009.03.056 19467744

[44] Looijmans-van den Akker I, van Delden JJ, Verheij TJ, van Essen GA, van der Sande MA, et al (2009) Which determinants should be targeted to increase influenza vaccination uptake among health care workers in nursing homes? Vaccine 27: 4724–4730. 10.1016/j.vaccine.2009.05.013 19450642

[45] Hofmann F, Ferracin C, Marsh G, Dumas R (2006) Influenza vaccination of healthcare workers: a literature review of attitudes and beliefs. Infection 34: 142–147. 16804657 10.1007/s15010-006-5109-5

[46] Cabana MD, Rand CS, Powe NR, Wu AW, Wilson MH, et al (1999) Why don't physicians follow clinical practice guidelines? A framework for improvement. JAMA 282: 1458–1465. 10535437 10.1001/jama.282.15.1458

[47] Tong A, Sainsbury P, Craig J (2007) Consolidated criteria for reporting qualitative research (COREQ): a 32-item checklist for interviews and focus groups. Int J Qual Health Care 19: 349–357. 17872937 10.1093/intqhc/mzm042

[48] Brewer NT, Chapman GB, Gibbons FX, Gerrard M, McCaul KD, et al (2007) Meta-analysis of the relationship between risk perception and health behavior: the example of vaccination. Health Psychol 26: 136–145. 17385964 10.1037/0278-6133.26.2.136

[49] Yaqub O, Castle-Clarke S, Sevdalis N, Chataway J (2014) Attitudes to vaccination: a critical review. Soc Sci Med 112: 1–11. 10.1016/j.socscimed.2014.04.018 24788111

[50] Lehmann BA, Ruiter RA, van Dam D, Wicker S, Kok G (2015) Sociocognitive predictors of the intention of healthcare workers to receive the influenza vaccine in Belgian, Dutch and German hospital settings. J Hosp Infect 89: 202–209. 10.1016/j.jhin.2014.11.009 25586987

[51] Harmsen IA, Mollema L., Kok G., Paulussen T.G.W., de Melker H.E., Ruiter R.A.C. (2014) A model of parents' intention to vaccinate their child. Maastricht: University of Maastricht.

[52] Baron-Epel O, Madjar B, Grefat R, Rishpon S (2013) Trust and the demand for autonomy may explain the low rates of immunizations among nurses. Hum Vaccin Immunother 9: 100–107. 10.4161/hv.22503 23108353 PMC3667920

[53] Lehmann BA, Ruiter RA, Wicker S, van Dam D, Kok G (2014) "I don't see an added value for myself": a qualitative study exploring the social cognitive variables associated with influenza vaccination of Belgian, Dutch and German healthcare personnel. BMC Public Health 14: 407 24775096 10.1186/1471-2458-14-407 PMC4021212

[54] Hakim H, Gaur AH, McCullers JA (2011) Motivating factors for high rates of influenza vaccination among healthcare workers. Vaccine 29: 5963–5969. 10.1016/j.vaccine.2011.06.041 21699950

[55] Poltorak M, Leach M, Fairhead J, Cassell J (2005) 'MMR talk' and vaccination choices: an ethnographic study in Brighton. Soc Sci Med 61: 709–719. 15899328 10.1016/j.socscimed.2004.12.014

[56] Healy CM, Rench MA, Baker CJ (2011) Implementation of cocooning against pertussis in a high-risk population. Clin Infect Dis 52: 157–162. 10.1093/cid/ciq001 21288837

[57] Juraskova I, Bari RA, O'Brien MT, McCaffery KJ (2011) HPV vaccine promotion: does referring to both cervical cancer and genital warts affect intended and actual vaccination behavior? Womens Health Issues 21: 71–79. 10.1016/j.whi.2010.08.004 21185992

[58] Dube E, Bettinger JA, Halperin B, Bradet R, Lavoie F, et al (2012) Determinants of parents' decision to vaccinate their children against rotavirus: results of a longitudinal study. Health Educ Res 27: 1069–1080. 10.1093/her/cys088 22907535

[59] Godin G, Vezina-Im LA, Naccache H (2010) Determinants of influenza vaccination among healthcare workers. Infect Control Hosp Epidemiol 31: 689–693. 10.1086/653614 20482373

[60] Harmsen IA, Mollema L., Kok G., Paulussen T.G.W., de Melker H.E., Ruiter R.A.C. (2014) A model of parents' intention to vaccinate their child. Maastricht: University of Maastricht.

[61] Leder S, Florack A, Keller J (2015) Self-regulation and protective health behaviour: how regulatory focus and anticipated regret are related to vaccination decisions. Psychol Health 30: 165–188. 10.1080/08870446.2014.954574 25137215

[62] Lagoe C, Farrar KM (2015) Are you willing to risk it? The relationship between risk, regret, and vaccination intent. Psychol Health Med 20: 18–24. 10.1080/13548506.2014.911923 24784419

[63] Liao Q, Wong WS, Fielding R (2013) How do anticipated worry and regret predict seasonal influenza vaccination uptake among Chinese adults? Vaccine 31: 4084–4090. 10.1016/j.vaccine.2013.07.009 23867015

[64] Chapman GB, Coups EJ (2006) Emotions and preventive health behavior: worry, regret, and influenza vaccination. Health Psychol 25: 82–90. 16448301 10.1037/0278-6133.25.1.82

[65] Aguilar-Diaz Fdel C, Jimenez-Corona ME, Ponce-de-Leon-Rosales S (2011) Influenza vaccine and healthcare workers. Arch Med Res 42: 652–657. 10.1016/j.arcmed.2011.12.006 22227045

[66] O'Neill O (2003) Some limits of informed consent. J Med Ethics 29: 4–7. 12569185 10.1136/jme.29.1.4 PMC1733683

[67] Nyhan B, Reifler J, Richey S, Freed GL (2014) Effective messages in vaccine promotion: a randomized trial. Pediatrics 133: e835–842. 10.1542/peds.2013-2365 24590751

[68] Hollmeyer H, Hayden F, Mounts A, Buchholz U (2013) Review: interventions to increase influenza vaccination among healthcare workers in hospitals. Influenza Other Respir Viruses 7: 604–621. 10.1111/irv.12002 22984794 PMC5781006

[69] Mercier H, Sperber D (2011) Why do humans reason? Arguments for an argumentative theory. Behav Brain Sci 34: 57–74; discussion 74–111. 10.1017/S0140525X10000968 21447233

[70] Larson HJ, Cooper LZ, Eskola J, Katz SL, Ratzan S (2011) Addressing the vaccine confidence gap. Lancet 378: 526–535. 10.1016/S0140-6736(11)60678-8 21664679

[71] Black S, Rappuoli R (2010) A crisis of public confidence in vaccines. Sci Transl Med 2: 61mr61. 10.1126/scitranslmed.3001738 21148125

[72] Beck U (1992) Risk Society Towards a New Modernity. London: Sage.

[73] Giddens A (1990) The Consequences of Modernity. Stanford: Stanford University Press 188 p.

[74] Peretti-Watel P, Raude J, Sagaon-Teyssier L, Constant A, Verger P, et al (2014) Attitudes toward vaccination and the H1N1 vaccine: poor people's unfounded fears or legitimate concerns of the elite? Soc Sci Med 109: 10–18. 10.1016/j.socscimed.2014.02.035 24681239

[75] Hobson-West P (2007) 'Trusting blindly can be the biggest risk of all': organised resistance to childhood vaccination in the UK. Sociol Health Illn 29: 198–215. 17381813 10.1111/j.1467-9566.2007.00544.x

[76] Blume S (2006) Anti-vaccination movements and their interpretations. Soc Sci Med 62: 628–642. 16039769 10.1016/j.socscimed.2005.06.020

[77] Gowda C, Dempsey AF (2013) The rise (and fall?) of parental vaccine hesitancy. Hum Vaccin Immunother 9: 1755–1762. 10.4161/hv.25085 23744504 PMC3906278

[78] Hak E, Schonbeck Y, De Melker H, Van Essen GA, Sanders EA (2005) Negative attitude of highly educated parents and health care workers towards future vaccinations in the Dutch childhood vaccination program. Vaccine 23: 3103–3107. 15837208 10.1016/j.vaccine.2005.01.074

[79] Smith PJ, Chu SY, Barker LE (2004) Children who have received no vaccines: who are they and where do they live? Pediatrics 114: 187–195. 15231927 10.1542/peds.114.1.187

